# Age‐at‐onset‐dependent effects of sulfur amino acid restriction on markers of growth and stress in male F344 rats

**DOI:** 10.1111/acel.13177

**Published:** 2020-06-22

**Authors:** Sailendra N. Nichenametla, Dwight A. L. Mattocks, Virginia L Malloy

**Affiliations:** ^1^ Animal Science Laboratory Orentreich Foundation for the Advancement of Science NY USA

**Keywords:** cysteine, ER stress, glutathione, hormesis, lifespan, methionine, trade‐offs, translational

## Abstract

Trade‐offs in life‐history traits are clinically and mechanistically important. Sulfur amino acid restriction (SAAR) extends lifespan. But whether this benefit comes at the cost of other traits including stress resistance and growth is unclear. We investigated the effects of SAAR on growth markers (body weight, IGF1, and IGFBP3) and physiological stresses. Male‐F344 rats were fed control (0.86% Met) and SAAR (0.17% Met) diets starting at 2, 10, and 20 months. Rats were injected with keyhole‐limpet‐hemocyanin (KLH) to measure immune responses (anti‐KLH‐IgM, anti‐KLH‐IgG, and delayed‐type‐hypersensitivity [DTH]). Markers of ER stress (FGF21 and adiponectin), detoxification capacity (glutathione [GSH] concentrations, GSH‐S‐transferase [GST], and cytochrome‐P_450_‐reductase [CPR] activities), and low‐grade inflammation (C‐reactive protein [CRP]) were also determined. SAAR decreased body weight, liver weight, food intake, plasma IGF1, and IGFBP3; the effect size diminished with increasing age‐at‐onset. SAAR increased FGF21 and adiponectin, but stress damage markers GRP78 and *Xbp1_s/us_* were unchanged, suggesting that ER stress is hormetic. SAAR increased hepatic GST activity despite lower GSH, but CPR activity was unchanged, indicative of enhanced detoxification capacity. Other stress markers were either uncompromised (CRP, anti‐KLH‐IgM, and DTH) or slightly lower (anti‐KLH‐IgG). Increases in stress markers were similar across all ages‐at‐onset, except for adiponectin, which peaked at 2 months. Overall, SAAR did not compromise stress responses and resulted in maximal benefits with young‐onset. In survival studies, median lifespan extension with initiation at 52 weeks was 7 weeks (*p* = .05); less than the 33.5‐week extension observed in our previous study with 7‐week initiation. Findings support SAAR translational studies and the need to optimize Met dose based on age‐at‐onset.

## Graphical abstract

Trade‐offs between the life‐history traits, growth, stress responses, and lifespan in sulfur amino acid restriction (SAAR). SAAR‐induced lifespan extension trade‐offs with growth but not stress responses. *Note*: Young, adult, and old represent onset of the intervention in 2‐month‐old, 10‐month‐old, and 20‐month‐old male F344 rats, respectively. SAAR – sulfur amino acid‐restricted diet (0.17% methionine without cysteine), CD – control diet (0.86% methionine without cysteine).

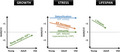

## 1 INTRODUCTION

Sulfur amino acid restriction (SAAR) is well documented to extend lifespan and confer several metabolic benefits (Ables, Hens, & Nichenametla, 2016). Much of these data are from studies in young rodent models under laboratory settings that do not impose environmental challenges. Empirical studies and aging theories suggest that lifespan extension causes trade‐offs in growth, reproduction, and stress resistance (McClure et al., 2014; Sung et al., 2017). Accordingly, SAAR‐induced increases in blood glutathione (GSH), plasma fibroblast growth factor 21 (FGF21), and adiponectin, and decrease in plasma insulin‐like growth factor 1 (IGF1) are concurrent with slow growth rates. As most of the studies initiated SAAR at 7–12 weeks, a period of rapid growth, it is unknown whether SAAR would be equally effective when initiated at later stages of life with reduced growth rates. Another less studied but important aspect of SAAR is whether the metabolic benefits are conferred at the cost of fitness, that is, response to environmental challenges, including immune response.

Nutritional requirements for methionine (Met) depend on life stage and physiological status (Otten, Hellwig, & Meyers, 2006). Young rats allocate 60% of dietary Met for growth and decrease this allocation exponentially with increasing body weight (Ishibashi & Kametaka, 1977; Shin, Owens, Pettigrew, & Oltjen, 1994). Considering that control diets in most SAAR studies contain 0.86% Met (and no cysteine [Cys]), restrictions of approximately 80% in rats (0.17% Met) and 86% in mice (0.12% Met) are essential for extending lifespan and inducing metabolic benefits. Previous studies report that lifespan extension, changes in FGF21, and adiposity were abrogated at restriction levels below these thresholds, that is, Met concentrations greater than 0.17% in rats and 0.12% in mice (Brown‐Borg et al., 2014; Forney, Wanders, Stone, Pierse, & Gettys, 2017). However, much of these data pertain to the onset of intervention in young animals. Accounting for the lower Met requirements in adult and old animals, we hypothesized that even 0.17% Met would be less efficient when initiated in mature adult and old rats.

The metabolism and requirements of sulfur amino acids (SAA) change during adverse physiological conditions such as infection, inflammation, and immune challenges (Malmezat et al., 2000). Met and Cys are precursors for the synthesis of GSH and several acute‐phase proteins (Litvak, Rakhshandeh, Htoo, & de Lange, 2013). Met flux through the transsulfuration pathway, which is essential for GSH synthesis, increases by 2.7‐folds in infected rats (Malmezat et al., 2000). Tracer studies in humans show that under optimal nutrition, transsulfuration is higher in the elderly than in young individuals, and when challenged with vaccination, the flux increases in both age**‐**groups (Mercier et al., 2006). These data suggest that when optimal nutrition is provided, old animals require more SAA than young animals to mount similar responses. A corollary of these findings implicates that under limited availability of SAA, immune responses mounted by adult and old animals would be lower than that mounted by young animals. But, whether this is true remains unknown. We hypothesized that the magnitude of SAAR‐induced changes and response to immune challenge depends on the age‐at‐onset (AAO) of the intervention. We tested our hypothesis by feeding male F344 rats starting at the ages of 2 (young), 10 (adult), and 20 months (old), with control (CD‐0.86% Met without Cys) and SAAR (0.17% Met without Cys) diets for nine weeks. These particular ages were selected as they represent different degrees of growth, maturity, and senescence (Turturro et al., 1999). In addition, lifespan studies were conducted in a separate cohort of male F344 rats, initiating SAAR at 52 weeks of age.

## 2 RESULTS

Statistical methods are described in detail in the Methods section. Statistical significances are expressed using different notations for error probability. *P_int_*—probability values for interaction between AAO and the dietary intervention; *P*—probability values for the effect of diet within each age‐group obtained by post hoc analysis; and *P_2t_*—probability values for the effect of diet within each age‐group obtained by two‐tailed Student's *t*‐test.

### 2.1 SAAR induces AAO‐dependent changes in morphometrics

**Figure 1:**
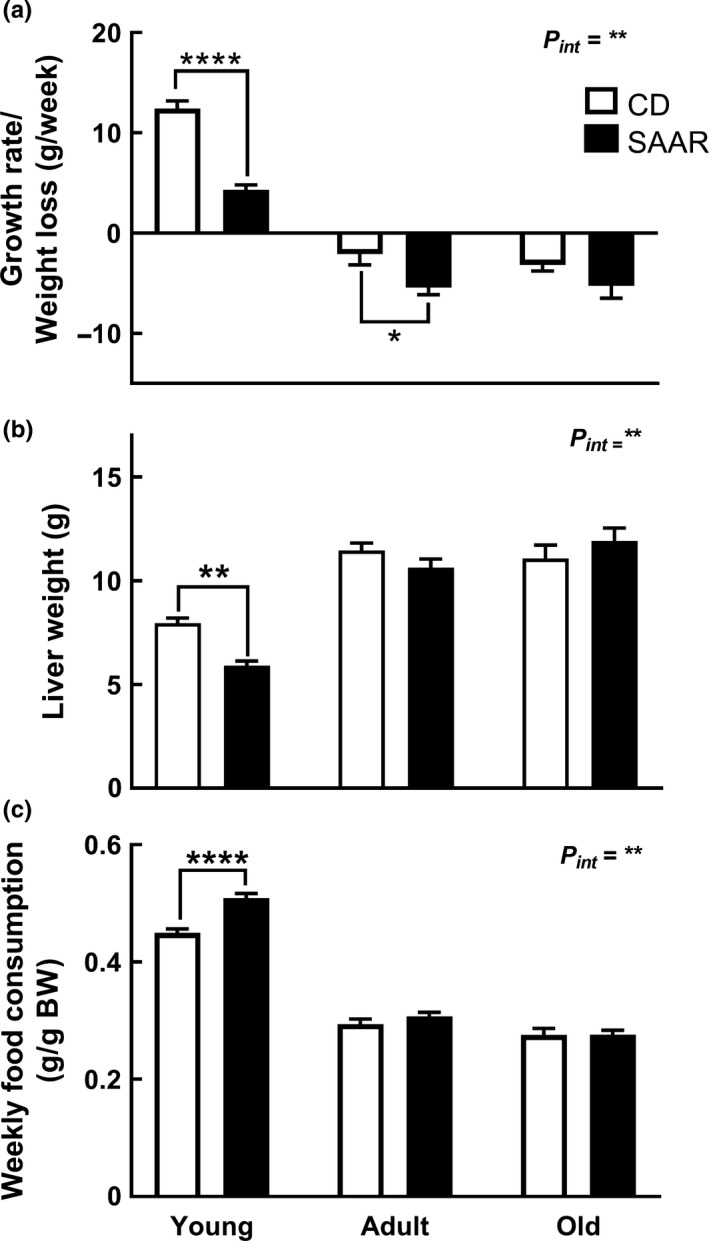
SAAR‐induced morphometric changes are dependent on age‐at‐onset. Control (CD ‐ 0.86% Met without Cys) and SAAR (0.17% Met without Cys) diets were fed to male F344 rats starting at ages 2 (young), 10 (adult), and 20 months (old) for nine weeks. (a) Changes in body weights (b) Changes in liver weights (c) Changes in food intake. *Note:* *n* = 5‐8/group; *P_int_—*interaction between age‐at‐onset and diet; error bars represent *SEM*; * *p* ≤ .05, ** *p* ≤ .01, **** *p* ≤ .0001

The effect of SAAR on body weight, liver weight, and food intake in young rats was similar to previous observations (Nichenametla, Mattocks, Malloy, & Pinto, 2017). A significant interaction between the diet and AAO was found for all three parameters (Figure 1, *P_int_* ≤ 0.01). Young rats grew three times slower on SAAR than on CD (Figure 1a, CD ‐ 12.43 g/week; SAAR ‐ 4.33 g/week; *p* ≤ .0001). Adult and old rats lost body weight on both diets. In adult rats, weight loss on SAAR was 2.5 times of that on CD (CD −2.08 g/week; SAAR −5.45 g/week; *p* ≤ .05). Old rats had similar weight loss on both diets. In young rats, SAAR decreased absolute liver weight (Figure 1b, CD ‐ 7.94 g; SAAR ‐ 5.90 g; *p* ≤ .01) but not in relation to body weight (data not shown). SAAR did not alter liver weight in adult and old rats. In young rats, food consumption in relation to body weight was significantly greater on SAAR than on CD (Figure 1c, SAAR ‐ 0.50 g/g body weight; CD – 0.45 g/g body weight; *p* ≤ .0001). SAAR did not affect food consumption in adult and old rats.

### 2.2 SAAR‐induced growth attenuation is independent of changes in growth hormone

**Figure 2:**
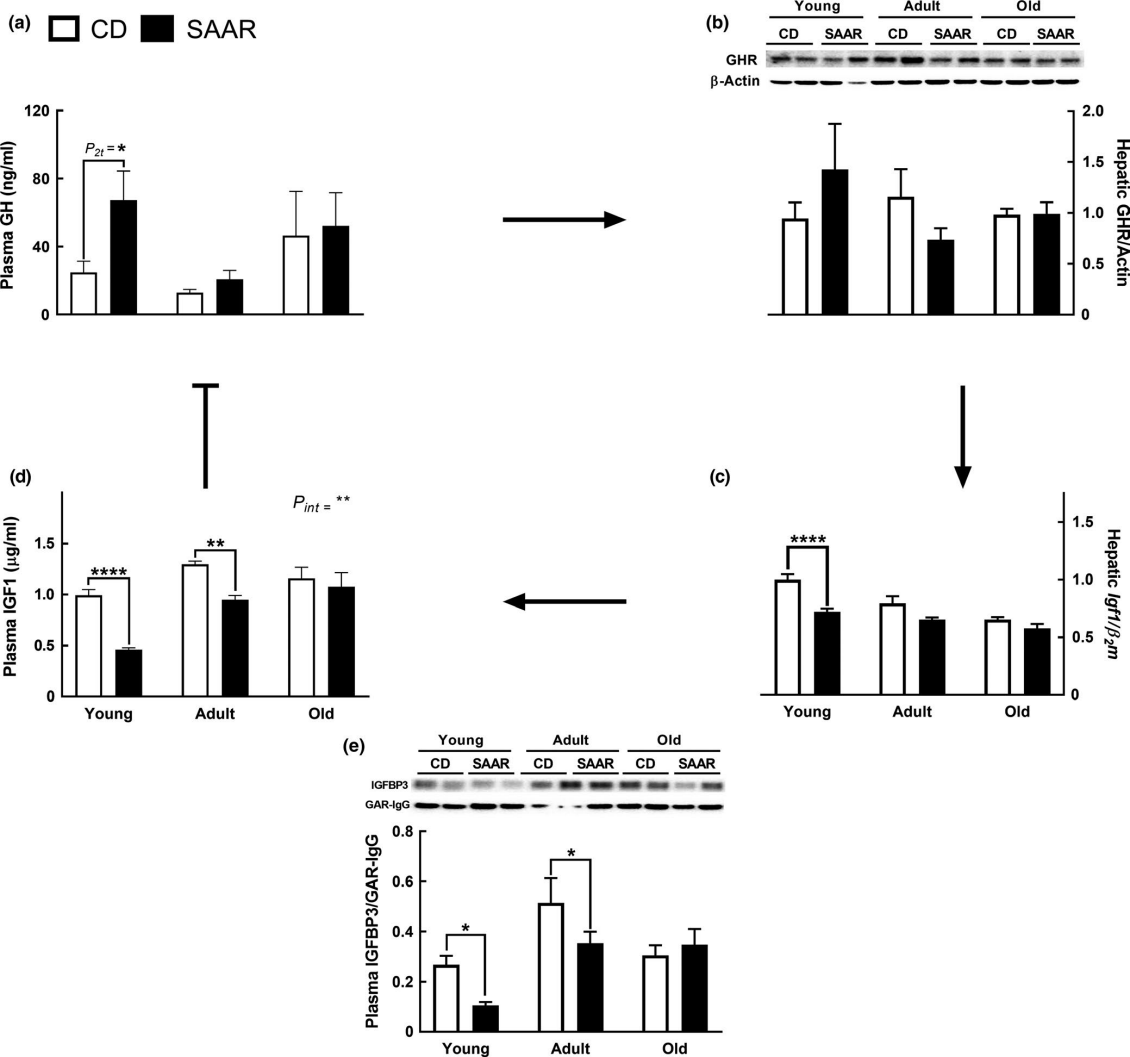
SAAR‐induced growth attenuation is independent of changes in growth hormone. Transcriptional and translational changes in GH/IGF1 signaling were determined in plasma and livers of young, adult, and old male F344 rats after nine weeks on SAAR diet. (a) Changes in plasma growth hormone. (b) Lack of changes in hepatic growth hormone receptor. C) Changes in hepatic *Igf1* mRNA. (d) Changes in plasma IGF1 concentrations. (e) Changes in plasma IGFBP3. *Note*: Arrows represent the feedback control of GH/IGF1 axis; GH*—*growth hormone; IGF1*—*insulin‐like growth factor1; GHR*—*growth hormone receptor; IGFBP3*—*IGF binding protein 3; *n* = 5‐8/group; error bars represent *SEM*; * *p* ≤ .05, ** *p* ≤ .01, **** *p* ≤ .0001; *P_2t_—p*‐values are from 2‐tailed Student's *t* test

In young rats, SAAR increased plasma growth hormone (GH) concentrations by 2.8‐fold (Figure 2a, CD – 24.8 ng/ml; SAAR – 67.3 ng/ml; *P_2t_* ≤ 0.05), but had no effect in adult and old rats. No differences were observed in hepatic growth hormone receptor (GHR) protein levels, regardless of AAO and diet (Figure 2b). Hepatic *Igf1* mRNA was lower in young rats on SAAR (Figure 2c, SAAR/CD – 0.72; *p* ≤ .0001), but unchanged in adult and old rats on SAAR. These changes were accompanied by similar and AAO‐dependent changes in plasma IGF1 levels (Figure 2d, *P_int_* < 0.01). SAAR decreased plasma IGF1 by 2‐folds in young rats (CD – 1 µg/ml; SAAR – 0.46 µg/ml; *p* ≤ .0001), 1.27‐fold in adult rats (CD – 1.29 µg/ml; SAAR – 0.94 µg/ml; *p* ≤ . 01), but had no effect in old rats (CD – 1.15 µg/ml; SAAR – 1.07 µg/ml). A significant negative correlation was found between IGF1 and GH levels in young animals on CD but not on SAAR (Figure S1a, CD: *r* −.75, *p* ≤ .05; SAAR: *r* −.07, *p* = .85). To confirm that higher plasma GH was not an artifact, we measured GH in young male F344 rats on SAAR from another study conducted under similar experimental conditions (Nichenametla et al., 2017). Although statistically not significant, results confirm a similar trend (Figure S1b). Regardless of age, all CD rats had similar plasma IGF1 levels. Our finding is in agreement with a previous study that reported no changes in IGF1 levels in rats that correspond to the age‐groups tested in the current study (2–20 months) (Breese, Ingram, & Sonntag, 1991). SAAR decreased the protein levels of plasma IGFBP3 in young rats (SAAR/CD – 0.39, *p* ≤ .05) and adult rats (SAAR/CD – 0.68, *p* ≤ .05) but not in old rats (Figure 2e).

### 2.3 SAAR antagonizes IGF1 by increasing transcription of IGF1 binding proteins

SAAR caused AAO‐dependent increases in mRNA expression of *Igfbp1* and *Igfbp4,* which antagonize the biological actions of IGF1 (Allard & Duan, 2018). SAAR increased *Igfbp‐1* only in young rats (Figure S2a, SAAR/CD = 2.2; *p* ≤ 0. 01*; P_int_* ≤ 0.05); and *Igfbp‐4* in both young (Figure S2b, SAAR/CD = 1.31; *p* ≤ .0001*, P_int_* ≤ 0.001) and adult rats (SAAR/CD = 1.39; *p* ≤ .0001). A larger increase was found for *Igfbp‐2* in young rats on SAAR (Figure S2c, *P_2t_* ≤ 0.001; SAAR/CD = 8.91).

### 2.4 SAAR does not compromise immune responses

**Figure 3:**
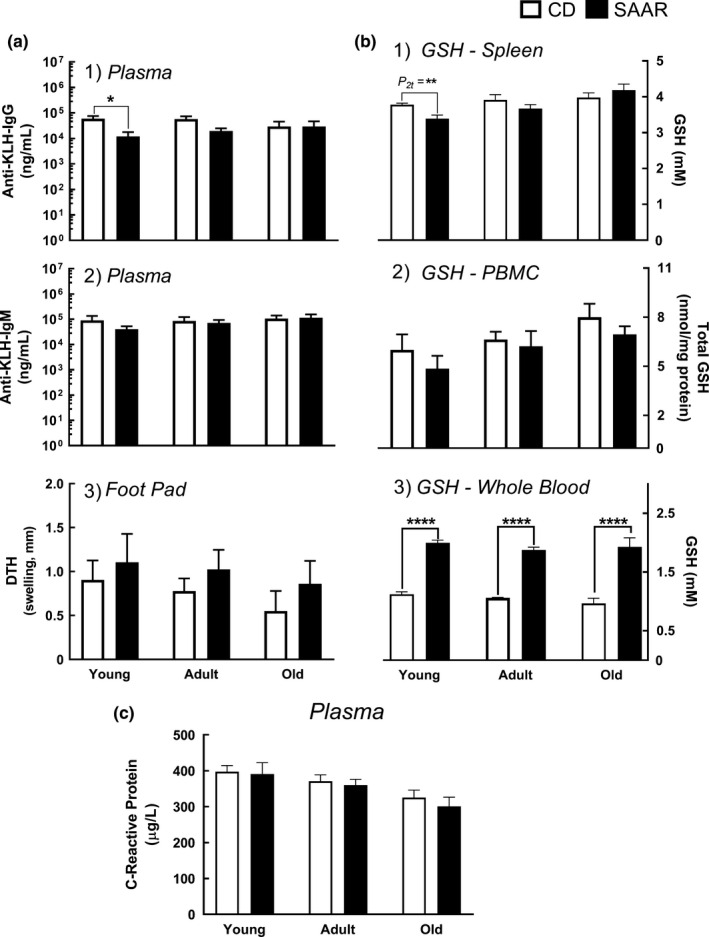
SAAR does not compromise immune stress responses. Primary (IgM), secondary (IgG), and delayed‐type hypersensitivity (DTH) immune responses against keyhole‐limpet‐hemocyanin (KLH), and chronic inflammation (C‐reactive protein [CRP]) were evaluated in young, adult, and old male F344 rats on SAAR diet for nine weeks. (a1‐a3) Immune responses, (a1) Plasma anti‐KLH‐IgG concentration, (a2) Plasma anti‐KLH‐IgM concentrations, (a3) Delayed‐type hypersensitivity response (DTH, determined by footpad swelling), (b1‐b3) Tissue‐specific changes in GSH, (b1) Changes in splenic GSH, (b2) Changes in PBMC GSH, (b3) Changes in whole blood GSH, (c) Lack of changes in plasma C‐reactive protein (CRP). *Note:* IgG, immunoglobulin G; IgM, immunoglobulin M; GSH, glutathione; *n* = 5‐8/group; error bars represent *SEM*; * *p* ≤ .05, ** *p* ≤ .01, **** *p* ≤ .0001; *P_2t_—* *P*‐values from 2‐tailed Student's *t*‐test

Humoral immune response was evaluated by injecting keyhole‐limpet‐hemocyanin (KLH) and determining anti‐KLH‐IgM and anti‐KLH‐IgG antibody levels. Some human studies reported the presence of anti‐KLH antibodies in unsensitized individuals (Moroz, Krygier, & Kotoulas, 1973). To rule out confounding effects, we determined anti‐KLH‐IgG antibodies from plasma collected before challenging with KLH, and the levels were undetectable (data not shown). Anti‐KLH‐IgM levels before sensitization were not determined as plasma was unavailable. SAAR decreased anti‐KLH‐IgG in young rats (Figure 3a1, SAAR/CD = 0.2; *p* ≤ .05) but not in adult and old rats; no changes were observed in the anti‐KLH‐IgM levels in any age‐group (Figure 3a2). Delayed‐type hypersensitivity (DTH) responses were similar regardless of diet and AAO (Figure 3a3). SAAR decreased splenic GSH content in young rats (Figure 3b1, SAAR/CD = 0.90; *P_2t_* ≤ 0.01) but not in adult and old rats. No changes were observed in GSH content of peripheral blood mononuclear cells (PBMC, Figure 3b2). SAAR caused AAO‐independent increases in whole blood GSH (Figure 3b3, SAAR/CD: young, adult, and old—1.78, 1.68, and 1.99; for all age‐groups *p* ≤ .0001), but did not alter plasma CRP concentrations (Figure 3c).

### 2.5 SAAR induces ER hormesis

**Figure 4:**
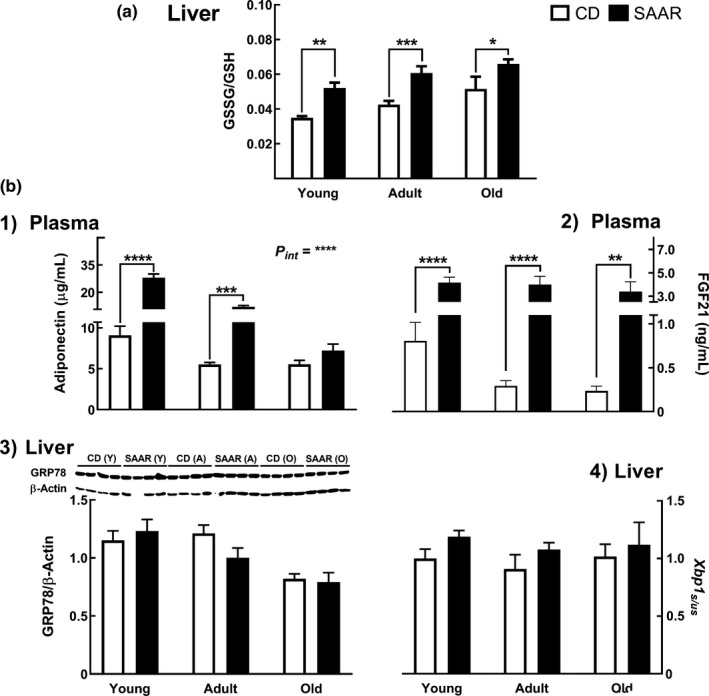
SAAR induces ER hormesis. Markers of ER stress were evaluated in young, adult, and old male F344 rats fed SAAR diet for nine weeks. (a) Increase in hepatic oxidative milieu, that is, GSSG/GSH b1‐b4) SAAR increased ER stress markers, (b1) Adiponectin (onset‐dependent) and (b2) FGF21 (onset‐independent), but did not alter markers of deleterious ER stress, that is, (b3) GRP78, and (b4) *Xbp1_s/us_*. *Note:* GSSG‐oxidized glutathione; GSH—reduced glutathione; FGF21—fibroblast growth factor 21; GRP78—glucose‐related protein 78; *Xbp1_s/us_*—ratio of spliced and unspliced transcripts of Xbox binding protein1; *n* = 5‐8/group; error bars represent *SEM*; *P_int_*—interaction between AAO and diet; * *p* ≤ .05, ** *p* ≤ .01, *** *p* ≤ .001; **** *p* ≤ .0001

SAAR increased the hepatic levels of GSSG/GSH, a marker of cellular redox status, in an AAO‐independent manner (Figure 4a, SAAR/CD—young, adult, and old—1.52, 1.46, and 1.29; for all age‐groups *P* at least ≤ 0.05). SAAR induced AAO‐dependent changes in plasma adiponectin (Figure 4b1, *P_int_* ≤ 0.0001). Adiponectin concentrations in SAAR‐treated rats were 3.2‐fold (*p* ≤ .0001) and 2.35‐fold (*p* ≤ .001) higher in young and adult rats, respectively, but similar in old rats. SAAR‐induced increase in plasma FGF21 concentration was AAO‐independent (Figure 4b2). Young, adult, and old rats on SAAR had 5.49‐fold, 14.61‐fold, and 15.28‐fold (for all age‐groups *P* at least ≤ 0.01) higher levels of FGF21, respectively. Although the effect of SAAR on FGF21 was AAO‐independent, the greater effect in adult and old rats compared to the effect in young rats is due to lower FGF21 levels in rats on control diet (adult/young – 0.32 and old/young – 0.26). SAAR did not induce ER stress as the protein levels of GRP78 (Figure 4b3) and splicing of X‐box binding protein 1 (Figure 4b4, *Xbp1_s/us_*) were not altered.

### 2.6 SAAR enhances detoxification capacity

**Figure 5:**
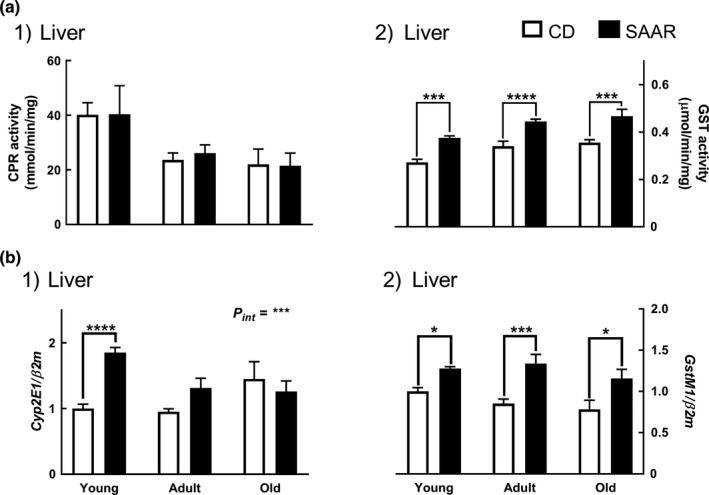
SAAR enhances detoxification capacity. Detoxification markers were evaluated in young, adult, and old male F344 rats fed SAAR diet for nine weeks. (a1) Lack of changes in the activity of phase‐1 detoxification enzyme, cytochrome‐P450‐reductase (CPR), (a2) Increase in the activity of phase‐2 detoxification enzyme, glutathione‐S‐transferase (GST). (b1) Increase in the mRNA expressions of cytochrome‐P_450_‐2E1 (*Cyp2E1*), and (b2) Glutathione‐S‐transferase M1 (*GstM1)*. *Note:* *n* = 5‐8/group; error bars represent *SEM*; * *p* ≤ .05, *** *p* ≤ .001, **** *p* ≤ .0001

SAAR did not compromise the CPR activity regardless of AAO (Figure 5a1), but increased GST activity across all ages (Figure 5a2, SAAR/CD: 1.38, 1.31, and 1.31 in young, adult, and old, for all age‐groups *P* at least ≤0.001). SAAR increased the expression of *Cyp2E1* only in young (Figure 5b1, SAAR/CD—1.8, *p* ≤ .0001), with a significant AAO‐diet interaction (*P_int_* ≤ 0.001). SAAR upregulated *GstM1* expression to a similar extent regardless of AAO (Figure 5b2, SAAR/CD: young—1.28; adult—1.57; and old—1.48; for all age‐groups *P* at least ≤0.05).

### 2.7 SAAR extends lifespan at adult‐onset

**Figure 6:**
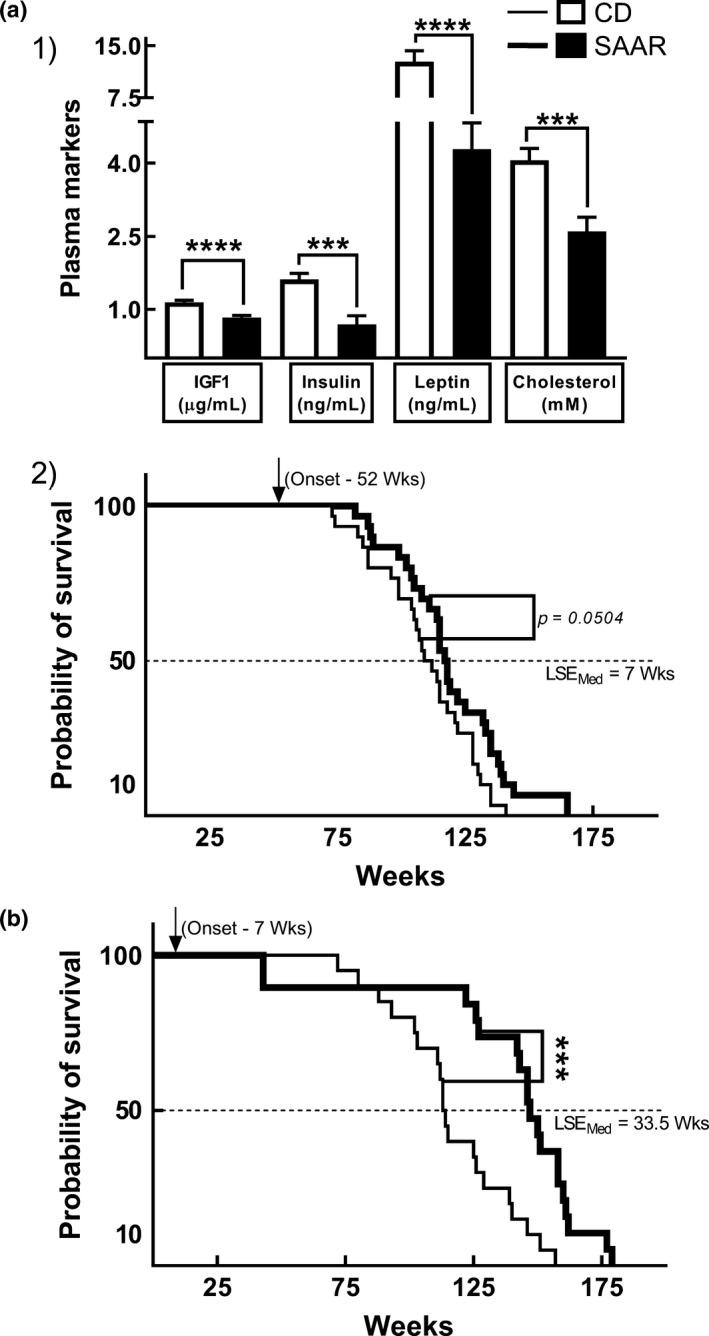
SAAR improves metabolic profile and extends lifespan at 52‐week onset. (a1) Changes in plasma metabolic profile six months after the 52‐week onset, (a2) Survival curves at 52‐week onset. (b) Survival curves at 7‐week onset. Compared to the initiation at 52 weeks, SAAR‐induced median lifespan extension at seven weeks initiation is approximately fivefold greater. *Note*: Data are derived from two different cohorts that are separate from the biomarker cohort. Survival curves in (b) are reproduced from a previous publication; n is different for each graph, (a1) 11‐12/group, (a2) 30/group, (b) 19‐20/group; Error bars in A1 represent *SEM*; all *p*‐values are from unpaired Student's *t* test; *** *p* ≤ .001; **** *p* ≤ .0001; LSE_Med_ indicates median lifespan extension

SAAR is effective even at 52‐week onset, as male F344 rats exhibited a better plasma metabolic profile (Figure 6a1, SAAR/CD: IGF1—0.72, insulin—0.43, leptin—0.34, and cholesterol—0.64; for all variables *P_2t_* at least ≤0.001). The median lifespan extension obtained at 52‐week onset was 7 weeks in male F344 rats (Figure 6a2, *p* = .0504). Although we detected a statistically significant lifespan extension, it is approximately fivefold lower than that obtained when initiated at the 7 weeks, that is, 33.5 weeks (Figure 6b, *p* ≤ .001). Survival curves for younger onset are reproduced from a published study conducted in the same laboratory under similar experimental conditions in the same model (Zimmerman, Malloy, Krajcik, & Orentreich, 2003).

## 3 DISCUSSION

We investigated two clinically important aspects of SAAR, that *is*, efficacy at different AAO and the effect on stress response markers. Decreases in body weight, liver weight, plasma IGF1, plasma IGFBP3, and increases in hepatic expressions of *Igfbp‐1*, *Igfbp‐2*, and *Igfbp‐4* were maximal in young rats, suggesting that SAAR inhibits growth. On the other hand, a comparison of survival curves indicates that the maximal lifespan extension is obtained with young onsets. Together, these data demonstrate that SAAR‐induced lifespan extension occurs at the cost of growth. On the contrary, depending on the type of stress, SAAR‐induced changes in stress markers indicate either enhanced response or no change. Some of the enhanced markers were AAO‐dependent (adiponectin and *Cyp2E1*), with maximal increases observed in young onset, while others were AAO‐independent (FGF21, *GstM1*, and GST activity). SAAR did not compromise primary immune response (anti‐KLH‐IgM), DTH, chronic low‐grade inflammation (plasma CRP concentrations), and phase‐1 detoxification capacity (hepatic CPR activity). Thus, SAAR‐induced lifespan extension does not tradeoff with stress responses. Despite resulting in maximal lifespan extension, translating SAAR in young children would be undesirable due to growth attenuation. Studies that focus on increasing the magnitude of AAO‐dependent changes in adult and old onsets would be beneficial for SAAR translation to humans.

In our study, the SAAR‐induced decrease in body weight was AAO‐dependent. The contributing mechanisms are likely to be very different in young and adult rats. Considering that young rats apportion a majority of dietary Met for growth, growth inhibition is likely the major contributor to the decrease in the body weights in young rats (Ishibashi & Kametaka, 1977; Shin et al., 1994). Since growth in mature animals is minimal or absent, these mechanisms are unlikely to be activated. Accordingly, several changes associated with growth inhibition, including, decrease in IGF1, IGFBP3, and increases in *Igfbp‐1, Igfbp‐2,* and *Igfbp‐4,* are either more pronounced or only occurred in young rats. The decrease in body weight in adult rats could be due to loss of tissue mass. Previous studies showed that the effect of SAAR on decreasing fat mass is much higher in adult mice than in young mice, while the magnitude of decrease in lean mass was similar in both young and adult mice (Lees et al., 2014). It is noteworthy that SAAR decreased body weights in adult rats despite not affecting food intake, suggesting that SAAR, in addition to preventing growth, induces active weight loss mechanisms.

The exact target of SAAR in GH/IGF1 signaling seems to be quite different from that in caloric restriction. SAAR decreased only IGF1 in young rats, whereas caloric restriction decreased both GH and IGF1 (Bonkowski et al., 2009). The dissociation between GH and IGF1 is also evident from the lack of correlation between the two in SAAR (*r* = −.07, *p* = .85), while a strong inverse correlation was found in CD (*r* = −.75, *p* = .03). In order to investigate whether the GH insensitivity is due to changes in GHR receptor biology, as occurs in Laron syndrome (due to GHR mutation) or during fasting (downregulation of GHR), we quantified hepatic GHR, but its levels were unchanged (Laron, 2015; Straus & Takemoto, 1990). The GH/IGF1 changes in SAAR seem to be initiated at the level of hepatic IGF1 transcription, resulting in lower levels of plasma IGF1. Our findings are consistent with prior observations where protein restriction in rats decreased the hepatic mRNA expression and plasma IGF1 levels despite exogenous administration of GH (Campbell, Johnson, King, Taverner, & Meisinger, 1990). Nutritional studies in pigs also show that the induction of IGF1 by GH requires a threshold level of dietary amino acids (Takenaka, Oki, Takahashi, & Noguchi, 2000). Taken together, SAAR‐induced changes in IGF1 signaling originate in the liver and are independent of GH. Recent studies show that GH has IGF1‐independent effects, particularly on the bone (Wu, Yang, & De Luca, 2015). Whether GH exerts such effects in SAAR remains unknown.

GSH, in addition to preventing oxidative damage, plays a critical role in various physiological stress responses (Rahman, Biswas, Jimenez, Torres, & Forman, 2005). Previous studies demonstrate that SAAR induces tissue‐specific changes in GSH concentrations (Richie et al., 2004). But the effect of these changes is not studied in detail, except on oxidative damage (Sanz et al., 2006). In this study, we quantified various stress markers that are known to respond to GSH concentrations (immune responses, ER stress, and detoxification capacity) from multiple tissues including liver, adipose tissue, spleen, and PBMC.

Contrary to our expectation and to previous reports of immune function enhancement by GSH, anti‐KLH‐IgM and DTH were unchanged, despite an increase in blood GSH by SAAR (Breitkreutz et al., 2000). We resolved this discrepancy by determining GSH concentration in immunogenic cells, PBMC and the immunogenic organ, spleen. The lack of changes in PBMC GSH concentrations explains the anti‐KLH‐IgM and DTH results (Figure S3a2,B2), while the lower concentration of anti‐KLH‐IgG in young rats might be a result of lower splenic GSH, where antibody class‐switching occurs (Figure S3a1,B1). The immune responses, therefore, reflected GSH changes in PBMC and spleen but not the changes in whole blood. The SAAR‐induced increase in blood GSH is different from increases observed with supplementation of either GSH or its precursor N‐acetylcysteine. Supplementation usually increases GSH levels in all compartments of blood including plasma, erythrocytes, and PBMC (Richie et al., 2015). The SAAR‐induced increase in blood GSH appears to be specific to erythrocytes, as we observed AAO‐independent increases in blood, 90% of which is erythrocytes, but no changes in PBMC. In addition, other investigators observed either no changes or slightly lower levels of GSH in plasma (Elshorbagy et al., 2011). In summary, SAAR did not appear to compromise the immune function. Additional studies are required to investigate the physiological relevance of higher GSH concentrations in erythrocytes but not other types of blood cells.

ER stress is implicated in increasing the secretion of FGF21 from liver and adiponectin from adipose tissue (Kyriakakis, Charmpilas, & Tavernarakis, 2017; Salminen, Kaarniranta, & Kauppinen, 2017). We previously demonstrated that SAAR induces ER stress‐like responses in the liver and asked if these responses are AAO‐dependent (Nichenametla et al., 2017). Of the two markers we quantified, changes in FGF21 were AAO‐independent, but adiponectin changes were AAO‐dependent. The AAO‐independent increase in both FGF21 and hepatic GSSG/GSH is consistent with our hypothesis that the higher plasma levels of FGF21 in SAAR are due to changes in oxidative milieu of liver, which induces ER stress. Depending on the magnitude of stress, ER stress responses can be detrimental. Unchanged GRP78 and *Xbp1_s/us_* levels, markers of deleterious ER stress, indicate that the hepatic ER stress is not severe but mild enough to induce beneficial cell protective mechanisms, that is, hormetic stress. Although we did not determine these markers in adipose tissue, we speculate that SAAR induces similar ER stress responses resulting in the increased secretion of adiponectin. However, we cannot explain the AAO dependency of changes in adiponectin. Overall, ER stress, initiated by a decrease in tissue GSH and an increase in the ratio of GSSG/GSH, might be a significant contributing mechanism by which SAAR extends lifespan.

Mice on SAAR have higher acetaminophen detoxification capacity (Miller et al., 2005). This seems paradoxical, as SAAR decreases the hepatic GSH which is required for detoxification of the toxic metabolite of acetaminophen, N‐acetyl‐p‐benzosemiquinone imine (NAPQI); acetaminophen *per se* is not toxic. Based on reports that Cyp450 enzymes, which convert acetaminophen to NAPQI, were repressed by protein‐deficient diets and are rescued by supplementation of either Met or Cys, we wondered whether the apparent increase in APAP resistance is due to active detoxification of NAPQI or decreased conversion of acetaminophen to NAPQI (Cho, Kim, Lee, & Kim, 1999). In addition, we questioned whether SAAR‐enhanced detoxification is specific to acetaminophen. For this, we determined the activities of enzymes that nonspecifically represent phase‐1 and phase‐2 detoxification systems, that is, CPR (which regulates the activity of all CYP450 enzymes by relaying electrons to them) and GST (using CDNB, a substrate on which all GST isoforms are active), respectively. While SAAR did not compromise the CPR activity, it enhanced the GST activity across all ages, demonstrating that the increased detoxification capacity may not be specific for acetaminophen. Besides, higher levels of *Cyp2E1,* which converts APAP to NAPQI and *GstM1,* which conjugates NAPQI with GSH, indicate that the SAAR‐induced acetaminophen resistance is due to active detoxification of NAPQI. We cannot address the molecular mechanisms for the enhanced detoxification in the current study. However, we speculate that SAAR‐induced increases in hepatic NRF2 levels might be the underlying mechanism (Nichenametla et al., 2017). The transcription factor, NRF2, enhances both phase‐1 and phase‐2 detoxification systems (Wu, Cui, & Klaassen, 2012).

Our study has some limitations. The primary objective of our study was to investigate representative markers of a broad range of physiological stresses but not detailed analyses of each type of stress. A clinically more relevant approach would be subjecting animals to multiple stressors. This requires challenging animals with multiple stressors such as lipopolysaccharide (inflammation), acetaminophen (toxicity), and antigens such as KLH (immunity) which could be life threatening, especially to the old group; hence, we challenged rats only with KLH. It is possible that animals respond differently when challenged simultaneously with multiple stressors. F344 rats are known for hyperactive hypothalamic–pituitary–adrenocortical axis (Marissal‐Arvy et al., 2007). Thus, additional studies are required to find if SAAR induces similar responses in other strains and species of laboratory models. While our findings demonstrate that young rats are more sensitive to SAAR than adult and old rats, it is quite possible that SAAR could be equally effective in adult and old rats upon intervening for durations longer than nine weeks. The difference in lag time to decrease body weights between young rats (immediate) and adult (5 weeks, Figure S3) indicates such a possibility. Thus, long‐term studies are required. Despite these limitations, our data provide valuable insights into the mechanisms and trade‐offs in the life‐history traits in SAAR, which are critical for its translation to humans.

Overall, we demonstrated that despite a decrease in the magnitude of AAO‐dependent changes, SAAR is effective in adult and old onsets and does not compromise stress responses. In addition, we also demonstrated that SAAR extends lifespan in adult onsets; however, the magnitude of the extension is less than that obtained in young onsets. Future studies should focus on optimizing the formulation of the SAAR diet to increase the efficacy in adult and old onsets. Considering the lower dietary requirement of SAA in mature animals, titrating down the Met concentration in the SAAR diet from 0.17% is a reasonable approach to increase the efficacy in adult and old onsets. Based on its promise in the preclinical studies in ameliorating the metabolic profile, a number of research groups, including ours, are moving toward clinical studies in diseased individuals (Dong, Sinha, & Richie, 2018; Gao et al., 2019). However, the benefits obtained in the few clinical studies conducted so far are not comparable to those in animal studies (Epner, Morrow, Wilcox, & Houghton, 2002; Olsen et al., 2020; Orgeron et al., 2014). It would be prudent for future preclinical and clinical studies to adjust the Met content of SAAR diets based on age‐, gender‐, and species‐specific nutritional requirements.

## 4 EXPERIMENTAL PROCEDURES

All animal procedures were approved by the Institutional Animal Care and Use Committee of the Orentreich Foundation for the Advancement of Science. Two cohorts of male F344 rats, one for biomarkers and the other for lifespan extension, were used. The biomarker cohort was used to study AAO‐specific effects on growth and stress markers. The lifespan cohort was used to monitor lifespan extension upon initiating SAAR at 52 weeks. The details of all the procedures performed on both cohorts are presented in the supporting information (Figure S4).

### 4.1 Animals and diets

#### 4.1.1 Biomarker cohort

Male F344 rats were purchased in two separate shipments from Charles River Laboratories (Wilmington, MA). The prospective old group of rats were obtained at 28–30 weeks of age (*n* = 17) and were maintained on Laboratory Rodent Diet 5001 (PMI Nutrition International, Brentwood, MO) and acidified water *ad libitum* until they reached 20 months of age. Three old rats died while being aged for the study. These deaths occurred on different days with the animals showing no overt signs of stress or discomfort. Young (7‐week‐old, *n* = 16) and adult (40‐week‐old, *n* = 16) animals were received when the old group approached 20 months of age. All animals were housed individually and maintained at a temperature of 20 ± 2°C, 50 ± 10% relative humidity, and a 12:12‐hr light–dark cycle. Rats from each age‐group were randomly assigned to feed on an isocaloric, chemically defined diet having the same amount of total amino acids (Research Diets, Inc., *n* = 8 for young and adult, *n* = 7 for old) for nine weeks. Met content in CD and SAAR diets was 0.86% and 0.17%, respectively. Glutamic acid was adjusted to offset the change in total amino acid content due to difference in Met concentration. Both diets were devoid of Cys (Table S1). During the study period, two old rats on CD and one on SAAR were found dead from unknown causes, and there were no overt signs of stress or discomfort. The diets were initiated when the young, adult, and old groups of rats were 2, 10, and 20 months old, respectively. All animals had access to the experimental diets and acidified water *ad libitum*. Body weight and food intake were measured weekly throughout the study. Interim blood at four weeks and seven weeks after initiating the diet was obtained to measure GSH content (whole blood and PBMC) and anti‐KLH‐IgM, respectively. Blood was also collected at the end of the study (9 weeks after initiating the diet). When animals were under isoflurane anesthesia (Primal Critical Care), blood was collected from the retro‐orbital plexus into EDTA vacutainer tubes (Becton‐Dickinson). Plasma and RBC were separated by centrifugation and stored at −80°C until analysis. Rats were euthanized with CO_2_ followed by decapitation. Liver and spleen were excised, immediately flash‐frozen, and stored at −80°C until analysis.

#### 4.1.2 Lifespan cohort

Male F344 rats were purchased from Taconic Farms (Germantown, New York, NY) at 9 –11 months of age. Due to the limited availability of aged animals, the animals were entered into the study in three batches, each of twenty, over a three‐month period. The rats were housed in pairs but maintained in the same animal facility and same housing conditions as used for the biomarker cohort. All the animals were fed CD diet (Ziegler Brothers, Table S1) from the time of receipt until they were entered into the study. At 12 months of age, the rats were weight‐matched and separated into two groups, one group continued to feed on CD, while the other was switched to the SAAR. Both groups stayed on these diets until death. Food and water were provided *ad libitum*. In order to eliminate the possibility of fighting, those animals who were not switched with their cagemate were individually housed. Every effort was made to age animals to their natural death; however, in some instances, if an animal was found to be moribund and deemed unable to survive for 24 hr, it was euthanized by CO_2_ asphyxiation. Six months after starting on the diet, blood was collected through retro‐orbital plexus, as described above.

### 4.2 Immune responses

Humoral and DTH responses were assessed during the last three weeks on the diet (Figure S4). Six weeks after the onset of diet, animals were sensitized by sub‐cutaneous injection of KLH (1 mg/0.2 ml saline, purity > 97%; EMD Millipore, Temecula, CA) into the interscapular skin fold. One week after sensitization, blood was collected from the retro‐orbital plexus under light anesthesia to measure primary (anti‐KLH‐IgM) immune responses. On the same day, the animals were then challenged with a second KLH injection. Two weeks after KLH challenge, blood was collected to measure secondary immune responses, that is, anti‐KLH‐IgG. Plasma was separated from blood and frozen at −80°C until analyzed. Anti‐KLH‐IgM and anti‐KLH‐IgG concentrations were determined by ELISA kits (Stellar Biotechnologies, Inc.) following the manufacturer's recommendations. DTH was determined after eight weeks on the diet, using foot‐pad swelling as an endpoint. Heat‐aggregated KLH was prepared as previously described, and 2 mg was injected subcutaneously into the right footpad (Nichenametla, South, & Exon, 2004). The swelling of the footpad was determined by the difference in thickness before and 24 hr after KLH injection. The thickness was measured using a digital micrometer following the manufacturer's instructions (Digi‐Micrometer, iGaging).

### 4.3 Glutathione determination

GSH levels in blood, liver, and spleen were determined by an enzymatic recycling method using 5,5‐dithiobis‐(2‐nitrobenzoic acid). Aliquots of blood, liver, and spleen were homogenized in 4–10 volumes of ice‐cold 5% (*w*/*v*) metaphosphoric acid (Sigma Chemical) and incubated on ice for 15 min. The homogenates were centrifuged at 10,000 *g* for 5 min at 4°C, and the acid‐soluble supernatant was used for GSH determination. Supernatants were further diluted based on the expected GSH concentrations in each tissue and used in the colorimetric assay as described previously (Nichenametla et al., 2017). To determine GSSG, a similar procedure was followed. However, free GSH was first derivatized with 2‐vinyl pyridine (Griffith, 1980). At four weeks on the diet, PBMC were isolated using Ficoll‐Paque density gradient media following manufacturer's recommendation (GE Healthcare and Life Sciences, Piscataway, NJ). To remove remnant erythrocytes, the final suspensions were gently dispersed and incubated for 5 min in 10 volumes of ammonium‐chloride potassium lysis buffer at room temperature. The isolates were centrifuged, and the buffer was discarded. The PBMC obtained were resuspended and washed in 100 µl of PBS. Pellets obtained after centrifuging and discarding the PBS were stored at −80°C until used. GSH in PBMC was determined by HPLC coupled with electrochemical detection at the Redox Core Facility of Oklahoma Nathan Shock Center of Excellence in the Biology of Aging (Oklahoma City, OK). On the day of assay, frozen PBMC were thawed and sonicated with 5% ice‐cold MPA. Homogenates were centrifuged at 15,000*g*, and supernatant fractions were filtered and injected into the pump. The details of HPLC chemistry are provided elsewhere (Rebrin, Kamzalov, & Sohal, 2003). Aliquots of PBMC homogenates were used for the determination of protein concentration by BCA assay. For all GSH assays, each sample was run in duplicate.

### 4.4 Plasma markers

#### 4.4.1 Biomarker cohort

ELISA kits (Table S2) were used to measure plasma GH, IGF1, FGF21, adiponectin, and CRP. Appropriate dilutions for each analyte were determined by testing representative samples from each group. All other procedures were followed according to the manufacturer's direction. All samples were run in duplicates.

#### 4.4.2 Lifespan cohort

After six months on diet, blood from overnight‐fasted animals was collected from the retro‐orbital plexus and processed for plasma as previously described (Malloy et al., 2006). IGF1, insulin, and leptin levels were quantified by radioimmunoassay according to the manufacturer's protocol (IGF1, DSL Webster; leptin and insulin, EMD‐Millipore). Plasma cholesterol was quantified using commercially available kits (Table S2). The principle of the assay involves enzymatic hydrolysis and oxidation followed by the development of indicator quinoneimine, in the presence of phenol and peroxidase. All samples were run in duplicates.

### 4.5 mRNA expression levels

Immediately after collecting, aliquots of the liver were incubated overnight with RNA*later* (Thermo Fisher Scientific) at 4°C. After discarding the RNA*later*, total RNA was extracted using TRIZOL (Thermo Fisher Scientific) as recommended by the manufacturer. RNA pellets were dissolved in water and tested for purity (260/280). Residual DNA was digested with DNase, and mRNA was reverse transcribed to cDNA using High Capacity cDNA Reverse Transcription Kits. Relative quantification of mRNA was performed by real‐time PCR using TaqMan assays that span exons (Thermo Fisher Scientific). All target gene assays were labeled with FAM, while the housekeeping gene assay beta‐2 microglobulin (*β2m*) was labeled with VIC. All samples were run in duplicates, and each replicate well contained primers for both the target and house‐keeping genes. Quantification for each gene was performed separately as singleplex. Fold changes in mRNA expression were calculated by normalizing the expression levels with those in young rats on CD. Assay information is presented in Table S3.

### 4.6 Protein quantification

#### 4.6.1 Western blots

Protein expression was determined by Western blotting. Briefly, flash‐frozen livers were homogenized with RIPA buffer using Potter–Elvehjem tissue homogenizer, and protein concentrations were determined by BCA assay. Plasma was directly used in the BCA assay. After SDS‐PAGE, proteins were transferred from gels to PVDF membranes (Bio‐Rad Laboratories, Inc.) and blocked with 5% nonfat dry milk (Bio‐Rad Laboratories, Inc.). After incubation with appropriately diluted primary antibodies, membranes were washed with TBST and incubated with secondary antibodies at room temperature. Band intensities were quantified with a ChemiDoc XRS + system after treating membranes with either with Clarity Western ECL Substrate (Bio‐Rad Laboratories, Inc.) or West Femto Maximum Sensitivity Substrate (Thermo Fisher Scientific). β‐Actin was used as the loading control for all proteins except IGFBP3. HRP‐conjugated goat anti‐rabbit IgG was used as loading control for the immunoprecipitated IGFBP3. The sample size ranged from 5‐8/group. Specific information on antibodies, incubation conditions, and dilutions is detailed in Table S4.

#### 4.6.2 Immunoprecipitation of IGFBP3 from plasma

Precleared plasma samples (350 µg protein/100 µl) were incubated overnight at 4°C with rabbit anti‐IGFBP3 antibody at 1:30 dilution (Abcam). 10 µl of a 50% protein A agarose bead slurry (Cell Signaling) was added to each sample and incubated with gentle rocking for 3 hr at 4°C. Following this, the immunocomplexes were centrifuged at 4,000*g* for 30 s at 4°C. The supernatants were discarded, and the pellets were washed five times on ice with 250 µl of TBS; each wash was followed by centrifugation at 4,000 *g* for 1 min at 4°C. The pellets were resuspended in 15 µl of 3X SDS sample buffer (187.5 mm Tris‐HCL, pH 6.8 at 25°C, 6% SDS, 30% glycerol, 150 mm DTT, 0.03% bromophenol blue), centrifuged for 30 s (4,000 *g*, 4°C), and heated in a boiling water bath for 10 min. The samples were centrifuged at 14,000 *g* for 1 min at 4°C; protein in the supernatant fraction was used in Western blots.

### 4.7 Enzyme activity

#### 4.7.1 Measurement of cytochrome P450 reductase activity in liver microsomes

Crude liver microsomes were isolated using a commercially available kit (Biovision). CPR activity was determined using another kit (Abcam). The principle of the assay is based on the coupling of NADPH oxidation by CPR with the reduction of a colorless probe to a colored product. NADPH is sourced *in situ* through the oxidation of glucose‐6‐phosphate to 6‐phospho‐D‐glucono‐1,5‐lactone by glucose‐6‐phosphatase dehydrogenase. The rate of the color change in the substrate was directly proportional to the CPR activity. Background activity due to the nonspecific reduction in the substrate by other NADPH‐dependent flavoproteins was accounted for with the use of an inhibitor in separate wells. Absorbance was read every 30 s for 30 min at 25°C. The activity was calculated from the linear portions of the curve and normalized to total protein in the reaction. CPR activity was expressed as mmol of NADPH oxidized/min/mg protein. All samples were run in duplicates.

#### 4.7.2 GST activity assay

GST activity was determined using a commercially available kit (Sigma Aldrich). The principle of the assay is based on the increase in absorbance of 1‐chloro‐2,4‐dinitrobenzene (CDNB), upon GST‐catalyzed conjugation with GSH. CDNB is a nonspecific substrate for several classes of GST isoforms, including α, µ, and π. Briefly, aliquots of the liver were homogenized in ice‐cold homogenization buffer (100 mm K_3_PO_4_, 0.2 mm EDTA, pH 7.0) using a Potter‐Elvehjem homogenizer. The homogenates were centrifuged at 10,000 *g* for 15 min at 4°C, and supernatant fractions were stored at −80°C until used. The assay was performed as recommended by the manufacturer. All samples were run in duplicate wells. GST activity was calculated based on the change in the absorbance over time and using the molar extinction coefficient of 3.5 mm ^−1^. Activity was normalized to the total amount of protein in the reaction and expressed as mmol of the conjugate formed/min/mg protein.

### 4.8 Statistical analysis

Data for the biomarker cohort were analyzed by two‐way analysis of variance considering AAO and diet as independent variables. The effect of SAAR in each age‐group was determined by using Sidak's correction for multiple comparisons. All data are expressed as mean ± standard error (*SEM*). Probability values for interaction between AAO and diet were reported using the notation *P_int_*. P‐values for *p* *ost‐hoc* comparison within each age‐group were reported using the notation *P*. In some cases, where diet‐induced differences within each age‐group were masked due to multiple comparisons and high variation in the old group, 2‐tailed Student's *t* test was used, and *p*‐values were indicated as *P_2t_* in the graphs. Associations between two variables were analyzed by Pearson's correlation test. Survival analyses for the lifespan cohort were conducted using the log‐rank test, and the unpaired Student's *t* test was used to analyze plasma marker data from lifespan cohort. In all cases, differences were considered statistically significant if *P* ≤ .05.

## CONFLICT OF INTEREST

None of the authors have any conflicts of interest.

## AUTHOR CONTRIBUTIONS

S. N. N. involved in study conception, study design, data analysis, data interpretation, and manuscript writing. V. L. M. involved in lifespan studies; animal husbandry and tissue harvesting for biomarker cohort. D. A. L. M. involved in animal husbandry and acquisition of all data from the biomarker cohort. All authors have reviewed and accepted the contents of this manuscript.

## Supporting information

Figure S1

Tables S1‐S4

Figure S2

Figure S3

Figure S4

## References

[acel13177-bib-0001] Ables, G. P. , Hens, J. R. , & Nichenametla, S. N. (2016). Methionine restriction beyond life‐span extension. Annals of the New York Academy of Sciences, 1363, 68–79. 10.1111/nyas.13014 26916321

[acel13177-bib-0002] Allard, J. B. , & Duan, C. (2018). IGF‐Binding Proteins: Why Do They Exist and Why Are There So Many? Front Endocrinol (Lausanne), 9, 117 10.3389/fendo.2018.00117 29686648 PMC5900387

[acel13177-bib-0003] Bonkowski, M. S. , Dominici, F. P. , Arum, O. , Rocha, J. S. , Al Regaiey, K. A. , Westbrook, R. , … Bartke, A. (2009). Disruption of growth hormone receptor prevents calorie restriction from improving insulin action and longevity. PLoS One, 4(2), e4567 10.1371/journal.pone.0004567 19234595 PMC2639640

[acel13177-bib-0004] Breese, C. R. , Ingram, R. L. , & Sonntag, W. E. (1991). Influence of age and long‐term dietary restriction on plasma insulin‐like growth factor‐1 (IGF‐1), IGF‐1 gene expression, and IGF‐1 binding proteins. The Journal of Gerontology, 46(5), B180–187. 10.1093/geronj/46.5.B180 1716275

[acel13177-bib-0005] Breitkreutz, R. , Pittack, N. , Nebe, C. , Schuster, D. , Brust, J. , Beichert, M. , … Dröge, W. (2000). Improvement of immune functions in HIV infection by sulfur supplementation: Two randomized trials. Journal of Molecular Medicine (Berlin), 78(1), 55–62. 10.1007/s001099900073 10759030

[acel13177-bib-0006] Brown‐Borg, H. M. , Rakoczy, S. G. , Wonderlich, J. A. , Rojanathammanee, L. , Kopchick, J. J. , Armstrong, V. , & Raasakka, D. (2014). Growth hormone signaling is necessary for lifespan extension by dietary methionine. Aging Cell, 13(6), 1019–1027. 10.1111/acel.12269 25234161 PMC4244257

[acel13177-bib-0007] Campbell, R. G. , Johnson, R. J. , King, R. H. , Taverner, M. R. , & Meisinger, D. J. (1990). Interaction of dietary protein content and exogenous porcine growth hormone administration on protein and lipid accretion rates in growing pigs. Journal of Animal Science, 68(10), 3217–3225. 10.2527/1990.68103217x 2254198

[acel13177-bib-0008] Cho, M. K. , Kim, Y. G. , Lee, M. G. , & Kim, S. G. (1999). Suppression of rat hepatic cytochrome P450s by protein‐calorie malnutrition: Complete or partial restoration by cysteine or methionine supplementation. Archives of Biochemistry and Biophysics, 372(1), 150–158. 10.1006/abbi.1999.1482 10562428

[acel13177-bib-0009] Dong, Z. , Sinha, R. , & Richie, J. P. Jr (2018). Disease prevention and delayed aging by dietary sulfur amino acid restriction: Translational implications. Annals of the New York Academy of Sciences, 1418(1), 44–55. 10.1111/nyas.13584 29399808

[acel13177-bib-0010] Elshorbagy, A. K. , Valdivia‐Garcia, M. , Mattocks, D. A. L. , Plummer, J. D. , Smith, A. D. , Drevon, C. A. , … Perrone, C. E. (2011). Cysteine supplementation reverses methionine restriction effects on rat adiposity: Significance of stearoyl‐coenzyme A desaturase. Journal of Lipid Research, 52(1), 104–112. 10.1194/jlr.M010215 20871132 PMC2999932

[acel13177-bib-0011] Epner, D. E. , Morrow, S. , Wilcox, M. , & Houghton, J. L. (2002). Nutrient intake and nutritional indexes in adults with metastatic cancer on a phase I clinical trial of dietary methionine restriction. Nutrition and Cancer, 42(2), 158–166. 10.1207/S15327914NC422_2 12416254

[acel13177-bib-0012] Forney, L. A. , Wanders, D. , Stone, K. P. , Pierse, A. , & Gettys, T. W. (2017). Concentration‐dependent linkage of dietary methionine restriction to the components of its metabolic phenotype. Obesity (Silver Spring), 25(4), 730–738. 10.1002/oby.21806 28261952 PMC5373958

[acel13177-bib-0013] Gao, X. , Sanderson, S. M. , Dai, Z. , Reid, M. A. , Cooper, D. E. , Lu, M. , … Locasale, J. W. (2019). Dietary methionine influences therapy in mouse cancer models and alters human metabolism. Nature, 572(7769), 397–401. 10.1038/s41586-019-1437-3 31367041 PMC6951023

[acel13177-bib-0014] Griffith, O. W. (1980). Determination of glutathione and glutathione disulfide using glutathione reductase and 2‐vinylpyridine. Analytical Biochemistry, 106(1), 207–212. 10.1016/0003-2697(80)90139-6 7416462

[acel13177-bib-0015] Huffman, D. M. , & Barzilai, N. (2009). Role of visceral adipose tissue in aging. Biochimica Et Biophysica Acta, 1790(10), 1117–1123. 10.1016/j.bbagen.2009.01.008 19364483 PMC2779572

[acel13177-bib-0016] Ishibashi, T. , & Kametaka, M. (1977). Methionine Requirements of Rats in Various Body Weights. Agricultural and Biological Chemistry, 41(9), 1795–1796. 10.1271/bbb1961.41.1795

[acel13177-bib-0017] Kyriakakis, E. , Charmpilas, N. , & Tavernarakis, N. (2017). Differential adiponectin signalling couples ER stress with lipid metabolism to modulate ageing in C. elegans. Scientific Reports, 7(1), 5115 10.1038/s41598-017-05276-2 28698593 PMC5505976

[acel13177-bib-0018] Laron, Z. (2015). Lessons from 50 Years of Study of Laron Syndrome. Endocr Pract, 21(12), 1395–1402. 10.4158/EP15939.RA 26401581

[acel13177-bib-0019] Lees, E. K. , Król, E. , Grant, L. , Shearer, K. , Wyse, C. , Moncur, E. , … Delibegovic, M. (2014). Methionine restriction restores a younger metabolic phenotype in adult mice with alterations in fibroblast growth factor 21. Aging Cell, 13(5), 817–827. 10.1111/acel.12238 24935677 PMC4331744

[acel13177-bib-0020] Litvak, N. , Rakhshandeh, A. , Htoo, J. K. , & de Lange, C. F. (2013). Immune system stimulation increases the optimal dietary methionine to methionine plus cysteine ratio in growing pigs. Journal of Animal Science, 91(9), 4188–4196. 10.2527/jas.2012-6160 23825332

[acel13177-bib-0021] Malloy, V. L. , Krajcik, R. A. , Bailey, S. J. , Hristopoulos, G. , Plummer, J. D. , & Orentreich, N. (2006). Methionine restriction decreases visceral fat mass and preserves insulin action in aging male Fischer 344 rats independent of energy restriction. Aging Cell, 5(4), 305–314. 10.1111/j.1474-9726.2006.00220.x 16800846

[acel13177-bib-0022] Malmezat, T. , Breuille, D. , Pouyet, C. , Buffiere, C. , Denis, P. , Mirand, P. P. , & Obled, C. (2000). Methionine transsulfuration is increased during sepsis in rats. American Journal of Physiology. Endocrinology and Metabolism, 279(6), E1391–1397. 10.1152/ajpendo.2000.279.6.E1391 11093928

[acel13177-bib-0023] Marissal‐Arvy, N. , Gaumont, A. , Langlois, A. , Dabertrand, F. , Bouchecareilh, M. , Tridon, C. , & Mormede, P. (2007). Strain differences in hypothalamic pituitary adrenocortical axis function and adipogenic effects of corticosterone in rats. Journal of Endocrinology, 195(3), 473–484. 10.1677/JOE-07-0077 18000309

[acel13177-bib-0024] McClure, C. D. , Zhong, W. , Hunt, V. L. , Chapman, F. M. , Hill, F. V. , & Priest, N. K. (2014). Hormesis results in trade‐offs with immunity. Evolution, 68(8), 2225–2233. 10.1111/evo.12453 24862588 PMC4282086

[acel13177-bib-0025] Mercier, S. , Breuille, D. , Buffiere, C. , Gimonet, J. , Papet, I. , Mirand, P. P. , & Obled, C. (2006). Methionine kinetics are altered in the elderly both in the basal state and after vaccination. American Journal of Clinical Nutrition, 83(2), 291–298. 10.1093/ajcn/83.2.291 16469986

[acel13177-bib-0026] Miller, R. A. , Buehner, G. , Chang, Y. , Harper, J. M. , Sigler, R. , & Smith‐Wheelock, M. (2005). Methionine‐deficient diet extends mouse lifespan, slows immune and lens aging, alters glucose, T4, IGF‐I and insulin levels, and increases hepatocyte MIF levels and stress resistance. Aging Cell, 4(3), 119–125. 10.1111/j.1474-9726.2005.00152.x 15924568 PMC7159399

[acel13177-bib-0027] Moroz, L. A. , Krygier, V. , & Kotoulas, A. O. (1973). Normal human IgG with antibody activity for keyhole limpet haemocyanin. Immunology, 25(3), 441–449. 4795397 PMC1423070

[acel13177-bib-0028] Nichenametla, S. N. , Mattocks, A. L. , Malloy, V. L. , & Pinto, J. T. (2017). Sulfur amino acid restriction–induced changes in redox‐sensitive proteins are associated with slow protein synthesis rates. Annals of NYAS (Healthy Aging), 1418(1), 80‐94. 10.1111/nyas.13556 29377163

[acel13177-bib-0029] Nichenametla, S. , South, E. , & Exon, J. (2004). Interaction of conjugated linoleic acid, sphingomyelin, and butyrate on formation of colonic aberrant crypt foci and immune functions in rats. J Toxicol Environ Health A, 67(6), 469–481. 10.1080/15287390490276494 14742093

[acel13177-bib-0030] Olsen, T. , Øvrebø, B. , Haj‐Yasein, N. , Lee, S. , Svendsen, K. , Hjorth, M. , … Vinknes, K. J. (2020). Effects of dietary methionine and cysteine restriction on plasma biomarkers, serum fibroblast growth factor 21, and adipose tissue gene expression in women with overweight or obesity: A double‐blind randomized controlled pilot study. Journal of Translational Medicine, 18(1), 122 10.1186/s12967-020-02288-x 32160926 PMC7065370

[acel13177-bib-0031] Orgeron, M. L. , Stone, K. P. , Wanders, D. , Cortez, C. C. , Van, N. T. , & Gettys, T. W. (2014). The impact of dietary methionine restriction on biomarkers of metabolic health. Prog Mol Biol Transl Sci, 121, 351–376. 10.1016/B978-0-12-800101-1.00011-9 24373243 PMC4049285

[acel13177-bib-0032] Otten, J. , Hellwig, J. , & Meyers, L. (2006). DRI values for indispensable amino acids by life stage and gender group Dietary Reference Intakes: The Essential Guide to Nutrient Requirements (pp. 459–465). Washington, DC: The National Academies of Press.

[acel13177-bib-0033] Rahman, I. , Biswas, S. K. , Jimenez, L. A. , Torres, M. , & Forman, H. J. (2005). Glutathione, stress responses, and redox signaling in lung inflammation. Antioxidants & Redox Signaling, 7(1–2), 42–59. 10.1089/ars.2005.7.42 15650395

[acel13177-bib-0034] Rebrin, I. , Kamzalov, S. , & Sohal, R. S. (2003). Effects of age and caloric restriction on glutathione redox state in mice. Free Radical Biology and Medicine, 35(6), 626–635. 10.1016/S0891-5849(03)00388-5 12957655 PMC2837076

[acel13177-bib-0035] Richie, J. P. Jr , Komninou, D. , Leutzinger, Y. , Kleinman, W. , Orentreich, N. , Malloy, V. , & Zimmerman, J. A. (2004). Tissue glutathione and cysteine levels in methionine‐restricted rats. Nutrition, 20(9), 800–805. 10.1016/j.nut.2004.05.009 15325691

[acel13177-bib-0036] Richie, J. P. Jr , Nichenametla, S. , Neidig, W. , Calcagnotto, A. , Haley, J. S. , Schell, T. D. , & Muscat, J. E. (2015). Randomized controlled trial of oral glutathione supplementation on body stores of glutathione. European Journal of Nutrition, 54(2), 251–263. 10.1007/s00394-014-0706-z 24791752

[acel13177-bib-0037] Salminen, A. , Kaarniranta, K. , & Kauppinen, A. (2017). Integrated stress response stimulates FGF21 expression: Systemic enhancer of longevity. Cellular Signalling, 40, 10–21. 10.1016/j.cellsig.2017.08.009 28844867

[acel13177-bib-0038] Sanz, A. , Caro, P. , Ayala, V. , Portero‐Otin, M. , Pamplona, R. , & Barja, G. (2006). Methionine restriction decreases mitochondrial oxygen radical generation and leak as well as oxidative damage to mitochondrial DNA and proteins. The FASEB Journal, 20(8), 1064–1073. 10.1096/fj.05-5568com 16770005

[acel13177-bib-0039] Shin, I. S. , Owens, F. N. , Pettigrew, J. E. , & Oltjen, J. W. (1994). Apportioning methionine requirements for maintenance versus growth of rats. Nutrition Research, 14(2), 229–239. 10.1016/S0271-5317(05)80382-3

[acel13177-bib-0040] Straus, D. S. , & Takemoto, C. D. (1990). Effect of fasting on insulin‐like growth factor‐I (IGF‐I) and growth hormone receptor mRNA levels and IGF‐I gene transcription in rat liver. Molecular Endocrinology, 4(1), 91–100. 10.1210/mend-4-1-91 2325671

[acel13177-bib-0041] Sung, E. J. , Ryuda, M. , Matsumoto, H. , Uryu, O. , Ochiai, M. , Cook, M. E. , … Hayakawa, Y. (2017). Cytokine signaling through Drosophila Mthl10 ties lifespan to environmental stress. Proc Natl Acad Sci U S A, 114(52), 13786–13791. 10.1073/pnas.1712453115 29229844 PMC5748187

[acel13177-bib-0042] Takenaka, A. , Oki, N. , Takahashi, S. I. , & Noguchi, T. (2000). Dietary restriction of single essential amino acids reduces plasma insulin‐like growth factor‐I (IGF‐I) but does not affect plasma IGF‐binding protein‐1 in rats. Journal of Nutrition, 130(12), 2910–2914. 10.1093/jn/130.12.2910 11110845

[acel13177-bib-0043] Turturro, A. , Witt, W. W. , Lewis, S. , Hass, B. S. , Lipman, R. D. , & Hart, R. W. (1999). Growth curves and survival characteristics of the animals used in the Biomarkers of Aging Program. Journals of Gerontology. Series A, Biological Sciences and Medical Sciences, 54(11), B492–501. 10.1093/gerona/54.11.B492 10619312

[acel13177-bib-0044] Wu, K. C. , Cui, J. Y. , & Klaassen, C. D. (2012). Effect of graded Nrf2 activation on phase‐I and ‐II drug metabolizing enzymes and transporters in mouse liver. PLoS One, 7(7), e39006 10.1371/journal.pone.0039006 22808024 PMC3395627

[acel13177-bib-0045] Wu, S. , Yang, W. , & De Luca, F. (2015). Insulin‐Like Growth Factor‐Independent Effects of Growth Hormone on Growth Plate Chondrogenesis and Longitudinal Bone Growth. Endocrinology, 156(7), 2541–2551. 10.1210/en.2014-1983 25910049

[acel13177-bib-0046] Zimmerman, J. A. , Malloy, V. , Krajcik, R. , & Orentreich, N. (2003). Nutritional control of aging. Experimental Gerontology, 38(1–2), 47–52. 10.1016/s0531-5565(02)00149-3 12543260

