# Supplementary figures and images for: Age‐at‐onset‐dependent effects of sulfur amino acid restriction on markers of growth and stress in male F344 rats
Source: Aging Cell. 2020 Jun 22;19(7):e13177. doi: 10.1111/acel.13177 (PMC7426777; doi:10.1111/acel.13177)

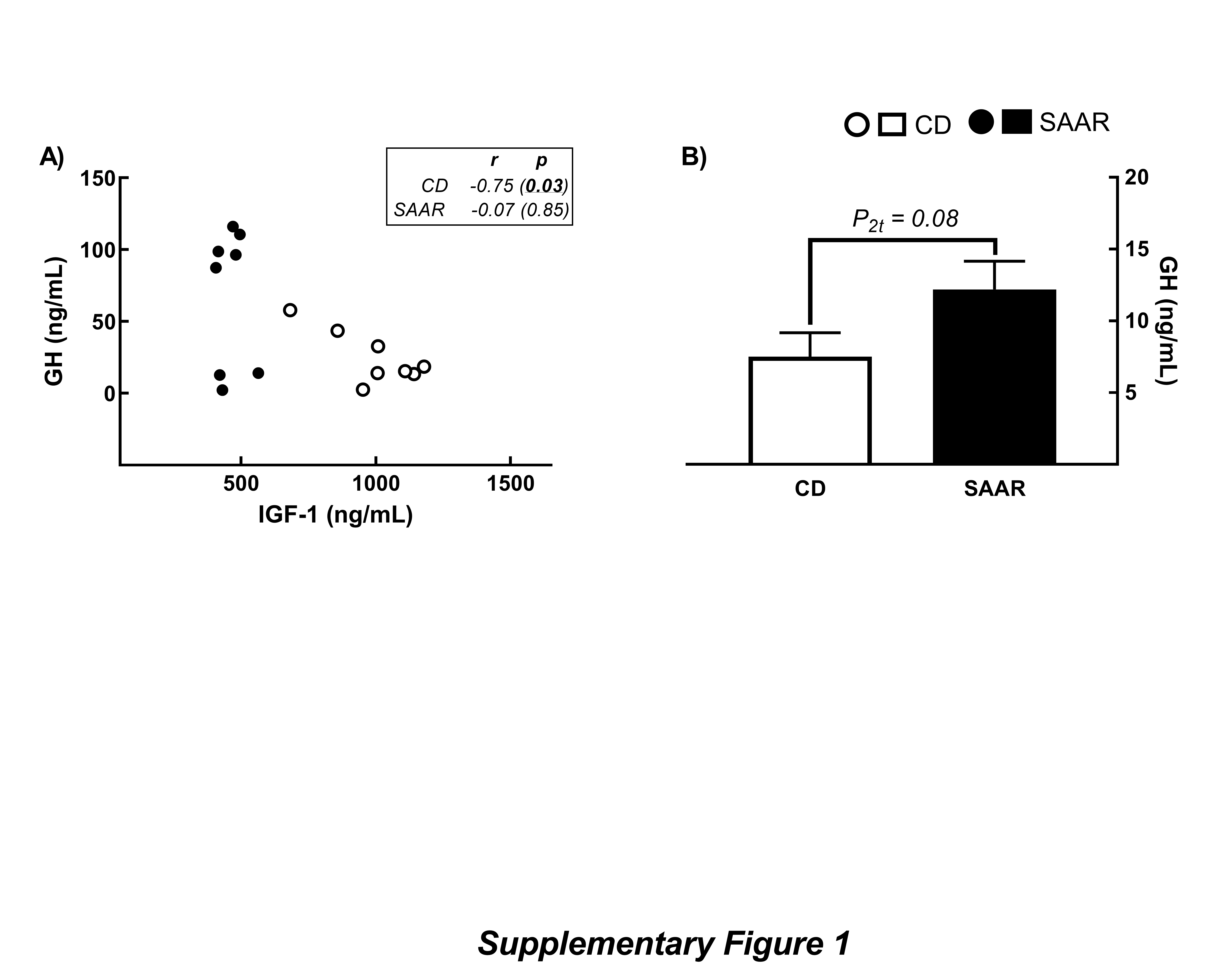

Supplement: Supplementary file 1 — Figure S1 [file ACEL-19-e13177-s001.tif]

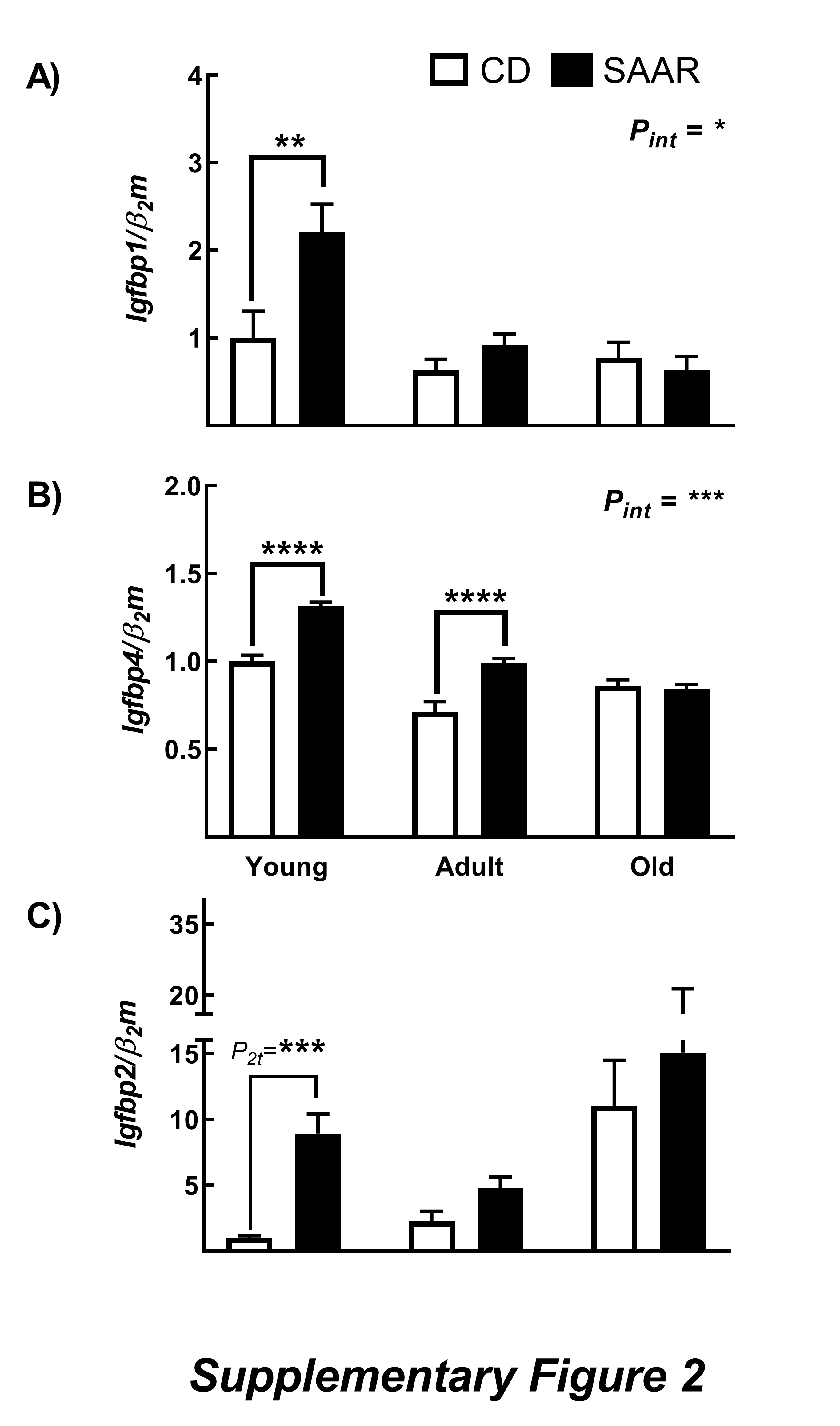

Supplement: Supplementary file 3 — Figure S2 [file ACEL-19-e13177-s003.tif]

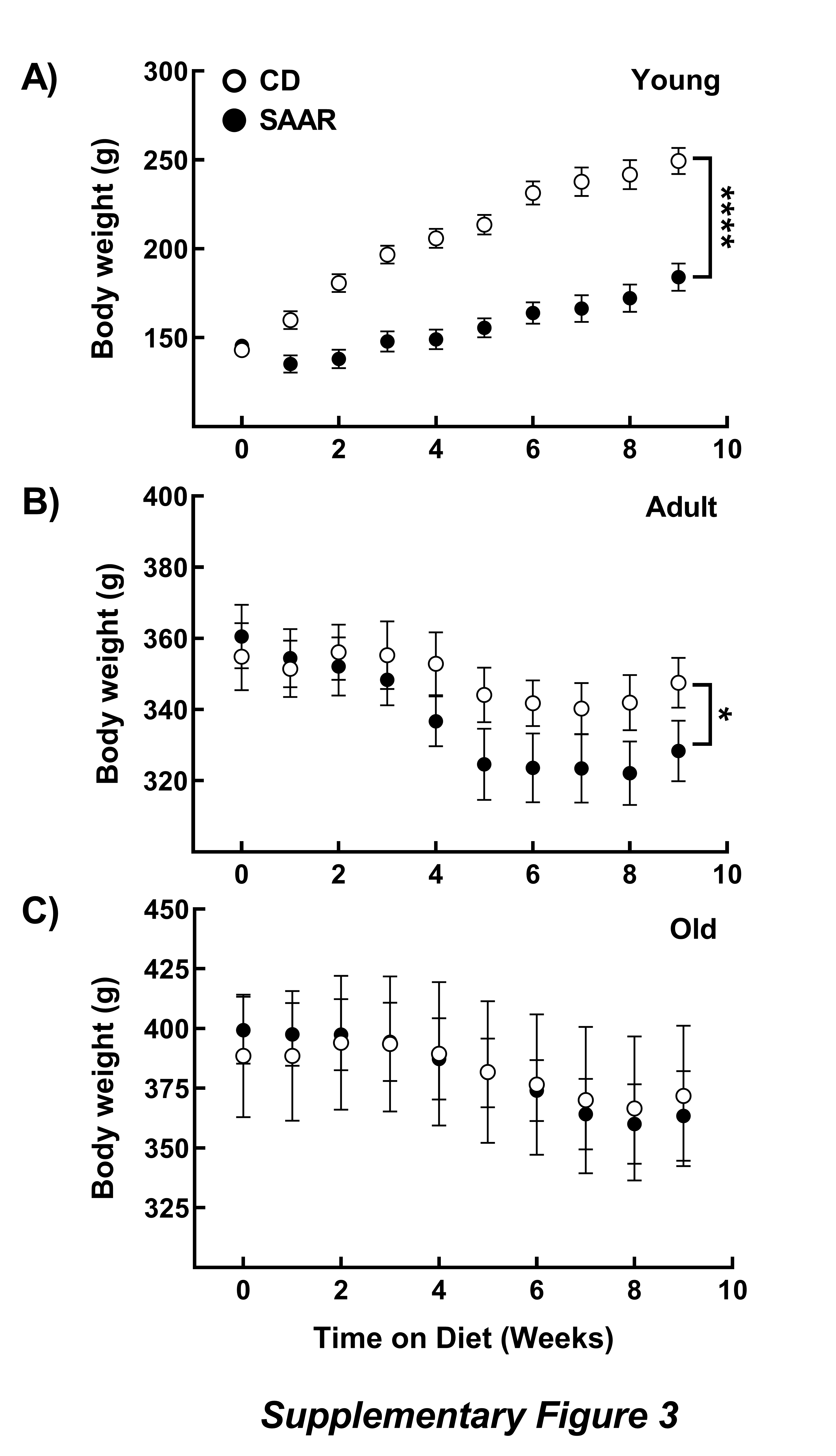

Supplement: Supplementary file 4 — Figure S3 [file ACEL-19-e13177-s004.tif]

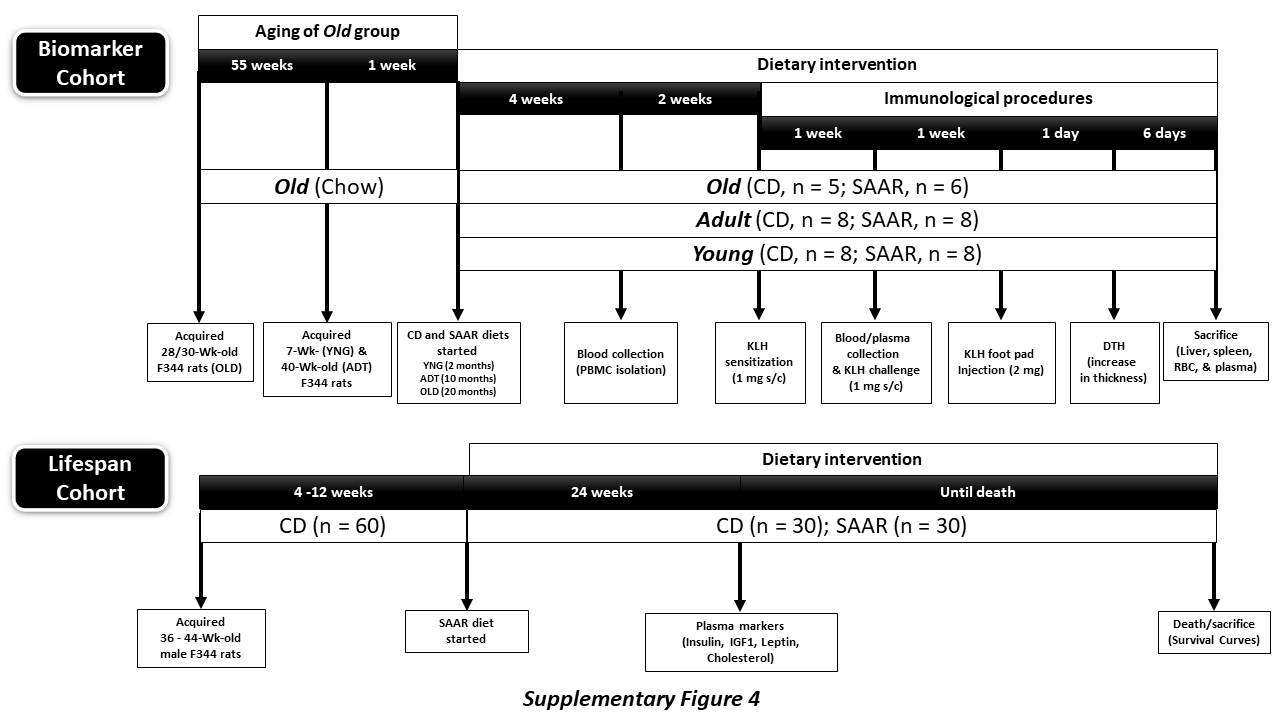

Supplement: Supplementary file 5 — Figure S4 [file ACEL-19-e13177-s005.tif]
