# Supplementary material for: Age‐at‐onset‐dependent effects of sulfur amino acid restriction on markers of growth and stress in male F344 rats
Source: Aging Cell. 2020 Jun 22;19(7):e13177. doi: 10.1111/acel.13177 (PMC7426777; doi:10.1111/acel.13177)
Supplement: Supplementary file 2 — Tables S1‐S4 [file ACEL-19-e13177-s002.docx]

1. ***Tables***

| **Supplementary Table 1. Dietary Composition** | |
| --- | --- |
| ***Macronutrient*** | ***Amount (Kcal/g)*** |
| Protein | 14 |
| Carbohydrate | 70 |
| Fat | 8 |
| ***Ingredient*** | ***Amount (g%)*** |
| L-Arginine | 1.12 |
| L-Histidine-HCl-H2O | 0.33 |
| L-Isoleucine | 0.82 |
| L-Leucine | 1.11 |
| L-Lysine | 1.44 |
| **DL-Methionine** | **0.86 (0.17)^a^** |
| L-Phenylalanine | 1.16 |
| L-Threonine | 0.82 |
| L-Tryptophan | 0.18 |
| L-Valine | 0.82 |
| **L-Glutamic Acid** | **2.7 (3.39) ^a^** |
| Glycine | 2.33 |
| Corn Starch | 36.11 **^b^** |
| Maltodextrin | 12.5 **^b^** |
| Sucrose | 20 |
| Cellulose | 5 |
| Corn Oil | 8 |
| Mineral Mix S10001 | 3.5 |
| Vitamin Mix V10001 | 1 |
| Choline bitartrate | 0.2 |
| **^a^** Numbers in parenthesis represent amounts in SAAR diet. CD and SAAR diets were isocaloric and contained same amount of amino acids.  **^b^** Corn starch and dextrin in diets used for lifespan cohort were 43.61 and 5, respectively | |
|  |  |

| **Supplementary Table 2. ELISA/RIA Kit Details** | | | |
| --- | --- | --- | --- |
| **Biomarker Cohort** | | | |
| **Plasma analyte** | **ELISA kit name (Catalog #)** | | **Manufacturer** |
| Growth hormone | Rat Growth Hormone (KRC5311) | | Thermo Fisher Scientific, Inc. |
| IGF1 | Mouse/Rat IGF1 (MG100) | | R&D Systems, Inc. |
| Anti-KLH-IgM | Rat Anti-KLH-IgM (ELI-02M) | | Stellar Biotechnologies, Inc. |
| Anti-KLH-IgG | Rat Anti-KLH-IgG (ELI-02G) | | Stellar Biotechnologies, Inc. |
| CRP | Rat C-Reactive Protein (ERCRP) | | Thermo Fisher Scientific, Inc. |
| Adiponectin | Rat Total Adiponectin/Acrp30 | | R&D Systems, Inc. |
| FGF21 | Mouse/Rat FGF21 (MF2100) | | R&D Systems, Inc. |
|  |  | |  |
| **Lifespan Cohort** | | | |
| **Plasma analyte** | | **RIA/Calometric kit name (Catalog #)** | **Manufacturer** |
| IGF1 | DSL Rat IGF-1 RIA (2900) | | DSL Webster, TX |
| Leptin | Rat Leptin RIA (RL-83K) | | EMD- Millipore, Billerica, MA |
| Insulin | Sensitive Rat Insulin RIA (SRI-13K) | | EMD- Millipore, Billerica, MA |
| Cholesterol | Total Cholesterol (CH200) | | Randox Laboratories, Kearneysville, WV |

| **Supplementary Table 3. TaqMan Assay Details** | |
| --- | --- |
| **Gene Name (symbol)** | **Assay ID** |
| Insulin-like growth factor-1 (*Igf1*) | Rn00710306_m1 |
| IGF-binding protein-1 (*Igfbp-1*) | Rn00565713_m1 |
| IGF-binding protein-2 (*Igfbp-2*) | Rn00565473_m1 |
| IGF-binding protein-4 (*Igfbp-4*) | Rn01464112_m1 |
| X-box binding protein-1 spliced (*Xbp1_s_*) | Rn03464499_m1 |
| X-box binding protein-1 unspliced (*Xbp1_us_*) | Rn04347716_s1 |
| Cytochrome P450 2E1 (*Cyp2E1*) | Rn00580624_m1 |
| GSH-S-transferase M1 (*GstM1*) | Rn00755117_m1 |
| Beta-2 Microglobulin (*β2M*) | Rn00560865_m1 |
| *All assays were obtained from Thermo Fisher Scientific* | |

| **Supplementary Table 4. Western Blotting Details** | | | | |
| --- | --- | --- | --- | --- |
| **Protein Name** (symbol) | **Primary Antibody** | | **Secondary Antibody** | |
|  | (Vendor /Catalog no) | (Dilution, incubation time@temperature) | (Vendor /Catalog no) | (Dilution, incubation time@temperature) |
| GHR | Invitrogen  PA5-79309 | 1:1000 in 5% milk, O/N @ 4^o^C | GAR-HRP (Cell Signaling Technology /#7074) | 1:7500 in 5% milk, 1 h @ RT |
|  |  |  |  |  |
| IGFBP3 | Abcam ab220429 | 1:1000 in 5% milk, 1.5 h @ RT | MAR-HRP Conformation-Specific (Cell Signaling Technology /#5127) | 1:7500 in 5% milk, 1 h @ RT |
|  |  |  |  |  |
| GAR-HRP IgG | GAR-HRP (Cell Signaling Technology #7074) | 1:7500 in 5% milk, 1 h @ RT | N/A | N/A |
|  |  |  |  |  |
| GRP78 | Novus Biologicals (AF4846) | 1:500 in 5% milk, O/N @ 4^o^C | DAG-HRP (Santa Cruz Biotechnology /sc-2020) | 1:10000 in 5% milk, 1 hr @ RT |
|  |  |  |  |  |
| β-Actin | Sigma A5441 | 1:20000 in 5% milk, 30 min @ RT | GAM-HRP (Bio-Rad /170-6516) | 1:20000 in 5% milk, 30 min @ RT |
| N/A – Not appllicable as the antibody was conjugated with HRP | | | | |
