# Supplementary material for: Age‐at‐onset‐dependent effects of sulfur amino acid restriction on markers of growth and stress in male F344 rats
Source: Aging Cell. 2020 Jun 22;19(7):e13177. doi: 10.1111/acel.13177 (PMC7426777; doi:10.1111/acel.13177)
Supplement: Supplementary file 6 [file ACEL-19-e13177-s006.docx]

**Supplementary Figure 1.** **SAAR induces hepatic growth hormone resistance in young onset**. **A)** Young rats on CD, but not SAAR, exhibited significant negative correlation between plasma GH and IGF1. **B)** Retrospective determination of plasma GH in the samples from a previously published study. *Note*: GH – growth hormone, IGF1 – Insulin-like growth factor 1; n=8/group; error bars represent SEM; *P_2t_* - *P*-values from 2-tailed Student’s t-test.

**Supplementary Figure 2. SAAR inhibits IGF1 biological activity by inducing the transcription of IGF binding proteins.** SAAR increased the hepatic mRNA expression of IGF binding proteins that antagonize IGF biological actions, including A) *Igfbp1*, B) *Igfbp4*, and C) *Igfbp2*. *Note:* n = 5-8/group; error bars represent SEM; **P* ≤ 0.05, **P* ≤ 0.01, **P* ≤ 0.001, *****P* ≤ 0.0001, *P_int_ –* interaction between AAO and diet; *P_2t_* - *P*-values from unpaired 2-tailed Student’s t-test.

**Supplementary Figure 3. Growth curves of biomarker cohort. A)** Young (2 month), **B)** Adult (10 month), and **C)** Old (20 month) male F344 rats were fed CD and SAAR diets. The decreasing effect of SAAR on body weights was immediate in young rats, required a lag time of five weeks in adult rats, and was absent in old rats. *Note:* n = 5-8/group; error bars represent SEM; **P* ≤ 0.05, *** *P* ≤ 0.0001.

**Supplementary Figure 4. A graphic representation of experimental design and procedures.** *Biomarker cohort*: Male F344 rats at different ages (young – 2 months, adult – 10 months, old – 20 months) were fed either control (CD - 0.86% Met without Cys) or SAAR diet (0.17% Met without Cys) for 9 weeks. Immunological procedures were performed during the last 3 weeks of the study. *Lifespan cohort*: 52-week-old male F344 rats were fed CD and SAAR diets until death. *Note*: PBMC – peripheral blood mononuclear cells, KLH – keyhole-limpet hemocyanin, DTH – delayed-type hypersensitivity.
